# Regulation of Nucleotide Metabolism with Nutrient‐Sensing Nanodrugs for Cancer Therapy

**DOI:** 10.1002/advs.202200482

**Published:** 2022-05-04

**Authors:** Xinye Wang, Wen Su, Yongbin Jiang, Fuhao Jia, Wenping Huang, Jie Zhang, Yue Yin, Hai Wang

**Affiliations:** ^1^ CAS Key Laboratory for Biomedical Effects of Nanomaterials & Nanosafety CAS Center for Excellence in Nanoscience National Center for Nanoscience and Technology Beijing 100190 China; ^2^ University of Chinese Academy of Sciences Beijing 100049 China; ^3^ Zhangjiakou First Hospital Zhangjiakou 075000 China

**Keywords:** biomolecular nanoparticles, cancer therapy, dietary modification, tumor metabolism

## Abstract

The continual growth of tumor cells requires considerable nutrient consumption. Methotrexate (MTX) is used to treat certain types of cancer by blocking the DNA and RNA productions through interfering one‐carbon metabolism and de novo purine and pyrimidine synthesis. However, treatment of MTX may cause many serious adverse effects, which hamper its clinical application. Herein, the authors synthesize ferrous ions, histidine, and MTX assembled nanoparticles (FHM) to deliver MTX at tumor site and enhance the sensitivity of tumor cells to MTX with histidine catabolism. Furthermore, fasting‐mimicking diet (FMD) is applied to intervene in the one‐carbon metabolism and enhance the cytotoxicity of MTX. Meanwhile, FMD treatment can significantly augment the cellular uptake and tumor accumulation of FHM nanoparticles. Due to the triple inhibitions of the one‐carbon metabolism, the proliferation of tumor cells is strongly disturbed, as which is highly replying on DNA and RNA production. Taken together, a 95% lower dose of MTX adopted in combined therapy significantly inhibits the growth of two types of murine tumors without evident systemic toxicity. This strategy may provide a promising nucleotide metabolism‐based nanomedicine for cancer therapy.

## Graphical abstract

Tumor cells show a higher activity of nucleotide anabolic pathway for uncontrolled proliferation. Triple inhibitions of one‐carbon metabolism and de novo pyrimidine or purine synthesis are achieved with ferrous ions, histidine, and methotrexate (MTX) assembled nanoparticles and fasting‐mimicking diet (FMD). With a 95% lower dose of MTX, the assembled nanoparticles efficiently inhibit the tumor growth together with FMD treatment.

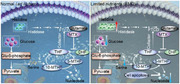

## 1 Introduction

Abnormal metabolism is a hallmark of malignant tumors.^[^ ^1^ ^]^ Tumor cells exhibit an altered nucleotide metabolism with a higher activity of nucleotide anabolic pathway compared to normal cells.^[^ ^2^ ^]^ Accordingly, a critical category of anticancer drugs is designed to reduce nucleotide synthesis in tumor cells such as methotrexate (MTX).^[^ ^2^ ^]^ A previous study reveals that histidine supplement significantly enhances the cytotoxicity of MTX by depletes tetrahydrofolate (THF) in tumor cells, suggesting histidine supplement is an effective complement to MTX‐based therapy.^[^ ^3^ ^]^ Unfortunately, these drugs cannot actively target tumor sites, which may induce a higher systemic toxicity.^[^ ^4^, ^5^ ^]^

Depriving essential nutrients is a valuable strategy to inhibit nucleotide metabolic pathways, such as glucose or protein restriction.^[^ ^6^, ^7^, ^8^ ^]^ Glucose produces 3‐phosphoglycerate, which is crucial for synthetizing serine.^[^ ^9^, ^10^ ^]^ Serine is the foremost provider of one‐carbon units and plays a vital role in the folate pathway.^[^ ^11^, ^12^ ^]^ Hence, controlled uptake of glucose and protein can be an adjunctive strategy for MTX‐based cancer therapy.^[^ ^13^ ^]^ However, the prolonged fasting proposes a high requirement to cancer patients and may result in other systemic damages.^[^ ^14^, ^15^ ^]^ Comparably, fasting‐mimicking diet (FMD) is a periodic diet that limits glucose and protein intake, which is a more appropriate option for patients.^[^ ^16^, ^17^ ^]^ Several clinical trials of FMD or fasting in patients showed no serious side effects and could be tolerated during the treatment.^[^ ^18^, ^19^ ^]^ Interestingly, FMD or fasting can reduce the potential side effects in cancer patients undergoing chemotherapy.^[^ ^20^, ^21^ ^]^ Moreover, FMD or fasting treatment augment the therapeutic outcomes of chemotherapy or reduce the incidence of drug resistance.^[^ ^22^ ^]^ Of note, the application of FMD or fasting in clinic should be carefully evaluated. Patients with significant weight loss or experiencing malnutrition may not suitable for this treatment.^[^ ^23^, ^24^ ^]^

**Figure 1:**
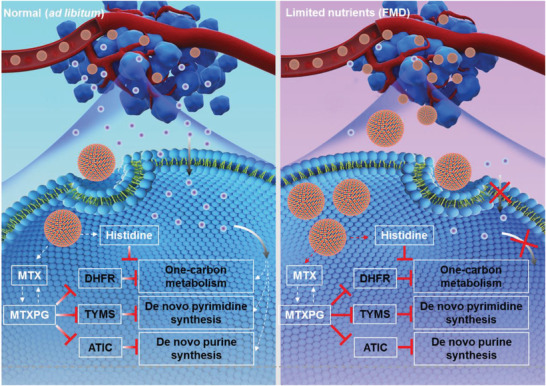
A schematic illustration of mechanisms of the enhanced anticancer capacity of nutrient‐sensed nanodrugs. MTX inhibits the functions of DHFR, TYMS, and ATIC, thereby decreasing one‐carbon metabolism and de novo pyrimidine or purine synthesis. Meanwhile, histidine metabolism further blocks the one‐carbon metabolism. As a result, histidine metabolism enhances the cytotoxicity of MTX by blocking the DNA and RNA synthesis. Moreover, FMD treatment can be used to enhance the cellular uptake and tumor accumulation of the FHM nanoparticles. Limited nutrients in the tumor microenvironment also help to decrease DNA and RNA synthesis for inhibiting the proliferation of tumor cells.

Taken together, we here reported a metal‐chelated histidine nanoparticle that could not only deliver MTX in the tumor site to decrease systemic toxicity through enhanced permeability and retention (EPR) effect,^[^ ^25^, ^26^ ^]^ but also enhance the cytotoxicity of MTX with histidine metabolism. Ferrous ion, histidine, and MTX were assembled to form MTX‐laden nanoparticles (FHM). After being delivered in tumor cells, MTX and its derivatives (methotrexate polyglutamate, MTXPG) inhibited the activities of dihydrofolate reductase (DHFR), aminoimidazole carboxamide adenosine ribonucleotide transformylase (ATIC), and thymidylate synthase (TYMS) to interfere one‐carbon metabolism, de novo purine, and pyrimidine synthesis, respectively (**Figure** **1**). Meanwhile, histidine metabolism depleted intracellular THF to augment the inhibition of one‐carbon metabolism (Figure 1). However, tumor cells could uptake various nutrients in the microenvironment to support the nucleotide pools, potentially reducing the cytotoxicity of MTX. In contrast, under FMD condition, the supply of nutrients was strongly blocked in the tumor microenvironment (Figure 1). As a result, our strategy strongly inhibited the DNA and RNA productions that are crucial for tumor cell proliferation. In vitro studies indicated that the combination of FHM nanoparticles and FMD treatments efficiently inhibited the proliferation of the three different kinds of tumor cells. Importantly, compared to a previously used dose,^[^ ^3^ ^]^ a 95% lower dose of MTX adopted in combined therapy (12.5 mg kg^−1^ body weight) significantly inhibited the growth of two types of murine tumors without evident systemic toxicity.

## 2 Results and Discussion

### 2.1 Characterizations of FHM Nanoparticles

**Figure 2:**
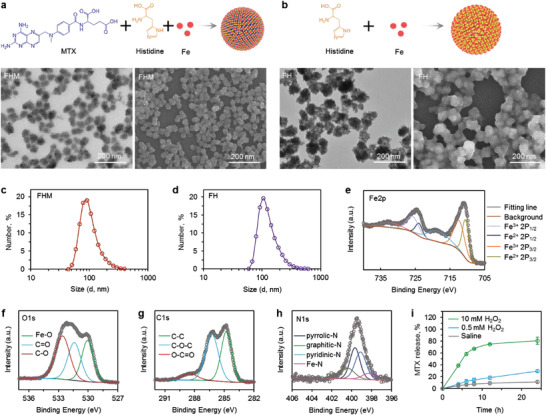
Characterization of FHM nanoparticles. a) A schematic illustration of the synthesize procedure for FHM nanoparticles. TEM (left) and SEM (right) images of FHM nanoparticles. Scale bar: 200 nm. b) A schematic illustration of the synthesize procedure for FH nanoparticles. TEM (left) and SEM (right) images of FH nanoparticles. Scale bar: 200 nm. c) The size distribution of the FHM or d) FH nanoparticles was determined by DLS at room temperature. e) XPS spectra for Fe2p, f) O1s, g) C1s, and h) N1s of FHM nanoparticles. i) The release profiles of FHM nanoparticles in hydrogen peroxide solutions with different concentrations of H_2_O_2_, indicating that MTX can be triggered by releasing from FHM nanoparticles under ROS condition. Error bars represent ± s.d. (*n* = 3).

To achieve synergistic effects, MTX, histidine, and ferrous ion were used to assemble the FHM nanoparticles (**Figure** **2**a). The morphology of prepared nanoparticles was investigated with transmission electron microscopy (TEM) and scanning electron microscope (SEM). The feeding ratios of MTX, histidine, and ferrous sulfate were first investigated. As shown in Figure 2a and Figure S1, Supporting Information, TEM images indicated that the feeding ratios of 1:10:5 exhibited uniform size distributions while aggregates were observed in others. SEM images further confirmed the uniform spherical nanostructures of FHM nanoparticles with a feeding ratio of 1:10:5, which was used in following studies. Nanoparticles were also formed by assembling histidine with ferrous ions (FH, Figure 2b), whereas no nanoparticles were observed if mixing ferrous ions with MTX (Figure S2, Supporting Information). The hydrodynamic size of FHM and FH nanoparticles were determined by dynamic light scattering (DLS) as shown in Figure 2d,e. The average size of FHM or FH nanoparticles is ∼104 or ∼130 nm, respectively. The UV–Vis absorbance was used to confirm the existence of MTX in FHM nanoparticles, showing a strong absorption peak at 370 nm (Figure S3, Supporting Information). This absorption peak was also observable in free MTX but not FH nanoparticles, revealing the successful assembling of MTX in FHM nanoparticles (Figure S3, Supporting Information). The encapsulation efficiency of MTX in FHM nanoparticles was 69.6 ± 1.2% at a feeding ratio of 1:10:5 (MTX: histidine: ferrous sulfate).

Next, the formation of FHM nanoparticles was investigated by X‐ray photoelectron spectroscopy (XPS). Figure 2e illustrates the Fe2p XPS spectra of the FHM nanoparticles. The binding energies of deconvoluted peaks confirmed the existence of Fe^2+^ and Fe^3+^ ions. Specifically, the peaks located at 710.5 (Fe 2p_3/2_) and 723.9 eV (Fe 2p_1/2_) were assigned to Fe^2+^,^[^ ^27^ ^]^ while the peaks centered at 712.3 (Fe 2p_3/2_) and 725.7 eV (Fe 2p_1/2_) were assigned to Fe^3+^.^[^ ^28^ ^]^ The peaks at 718.6 and 732.1 eV were the satellite peaks.^[^ ^29^, ^30^ ^]^ As illustrated in Figure 2f, the O1s XPS spectra was deconvoluted into three different peaks. The peak centered at 530.1 eV was assigned to Fe—O, suggesting coordinated interaction between ferrous ions and MTX or histidine.^[^ ^31^ ^]^ The peaks located at 531.4 and 532.6 eV were assigned to C═O and C—O,^[^ ^32^, ^33^ ^]^ respectively. Figure 2g displayed the C1s XPS spectra of FHM nanoparticles, which was potentially deconvoluted into three peaks. The peak at 284.8 eV was assigned to the C—C group,^[^ ^34^ ^]^ while the peak centered at 286.3 eV could be assigned to C—O—C group.^[^ ^35^ ^]^ The peak at 288.5 eV was ascribed to O—C═O,^[^ ^36^ ^]^ which originated from the carboxylic group in histidine and MTX. As for N1s XPS spectra, the peaks located at 399.1, 399.7, and 400.4 eV were assigned to pyridinic‐N, pyrrolic‐N, and graphitic‐N, respectively,^[^ ^37^, ^38^ ^]^ which is in good agreement with the molecular structure of histidine and MTX (Figure 2h). Importantly, the peak at 398.3 eV was assigned to Fe—N, indicating ferrous ion were coordinated with N elements in MTX or histidine to form the nanoparticles (Figure 2h).^[^ ^39^ ^]^ This is further confirmed with FTIR spectra. As shown in Figure S4, Supporting Information, FTIR absorbance bands in 1637 cm^−1^ of histidine and MTX referred to the carboxyl groups shifted to 1608 cm^−1^, probably due to the strong intramolecular coordination bonding between carboxyl group and ferrous irons. Imidazole absorbance band of histidine in 1496 cm^−1^ shifted to 1504 cm^−1^, indicating the formation of strong intramolecular coordination between imidazole and ferrous irons.

Additionally, it is important to release therapeutic agents at tumor site to induce cytotoxicity. We found that the FHM nanoparticles were sensitive to reactive oxygen species (ROS), which is abundant in tumor cells or the tumor microenvironment.^[^ ^40^ ^]^ As shown in Figure 2i, ROS strongly triggered the release of MTX from FHM nanoparticles with a concentrations‐depended behavior. Moreover, previous studies confirmed that FMD treatment could induce anti‐Warburg effect and increase ROS production in tumors,^[^ ^41^ ^]^ which might result in an efficient drug releasing at tumor site. The morphology of FHM nanoparticles was then checked with TEM images. As shown in Figure S5, Supporting Information, the nanostructure was gradually disassembled after being exposed to ROS, further confirming the ROS‐responsiveness of FHM nanoparticles.

### 2.2 In Vitro Antitumor Capacity of FHM Nanoparticles

**Figure 3:**
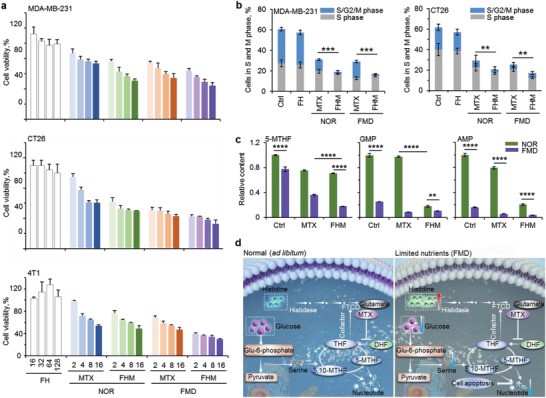
In vitro antitumor capacity of FHM nanoparticles. a) Cell viability of MDA‐MB‐231, CT26, and 4T1 tumor cells treated with FH nanoparticles, free MTX, or FHM nanoparticles with or without FMD pretreatment at MTX concentrations of 2, 4, 8, and 16 µg mL^−1^. Error bars represent ± s.d. (*n* = 3). b) Cell cycle analysis of MDA‐MB‐231 and CT26 tumor cells treated with FH nanoparticles, free MTX, or FHM nanoparticles with or without FMD pretreatment at an MTX concentration of 16 µg mL^−1^. Error bars represent ± s.d. (*n* = 3). Significant differences were detected by one‐way ANOVA with Tukey's multiple comparisons test, **p < 0.01, ***p < 0.001. c) Detection of 5‐MTHF, GMP, and AMP amounts in MDA‐MB‐231 cells treated with free MTX or FHM nanoparticle, with or without FMD treatment. Error bars represent ± s.d. (*n* = 3). Significant differences were detected by two‐way ANOVA with Tukey's multiple comparisons test, **p < 0.01, ****p < 0.0001. d) The metabolic pathway of MTX, histidine, and glucose. MTX inhibits the activity of DHFR, which catalyzes the reduction of DHF to THF. The metabolism of histidine to amino acid glutamate requires histidase, FTCD, and others, leading to the increased consumption of THF. Glucose catabolism reduction contributes to cellular THF depletion by decreasing the de novo synthesis of serine, as the *β*‐carbon on serine can be transferred to THF by SHMT.

Two types of murine tumor cells (4T1 and CT26) and one type of human tumor cells (MDA‐MB‐231) were used to test the antitumor capacity of FHM nanoparticles. We utilized starvation pretreatment to mimic the nutrient‐level changes during FMD treatment. Tumor cells were cultured in low‐glucose (0.5 g L^−1^) and low‐fetal bovine serum (FBS, 1%) medium for 24 h before exposing to various drug formulations. For normal condition (NOR), tumor cells were cultured with normal medium (2 g L^−1^ of glucose and 10% of FBS). As shown in **Figure** **3**a, FH nanoparticles (without MTX) did not induce cytotoxicity to all three types of tumor cells at different concentrations used for FHM nanoparticles. Importantly, FHM nanoparticles manifested higher cytotoxicity to MDA‐MB‐231, CT26, and 4T1 cells than free MTX (Figure 3a), suggesting that histidine supplement enhanced the cytotoxicity of MTX. Moreover, the cytotoxicity of free MTX was significantly increased when combined with FMD treatment (Figure 3a). This is probably because the limited nutrients in medium inhibited the DNA and RNA synthesis in tumor cells, generating synergistic effects with MTX. Overall, the best antitumor results were achieved by combining FHM nanoparticles and FMD treatment for all three types of tumor cells.

Since the DNA synthesis is directly related to the cell cycle,^[^ ^42^ ^]^ we then examined the cell cycle of MDA‐MB‐231 and CT26 cells using propidium iodide staining following various treatments. DNA replication takes place during the S phase. Afterward, tumor cells enter the G2 phase in which RNA, proteins, other macromolecules necessary for multiplication of cell organelles, and spindle formation are synthesized. Cell proliferation is started by going into the mitotic phase (M phase). Hence, tumor cells located in S, G2, and M phases should be decreased if limiting the productions of DNA and RNA. Indeed, shown in Figure 3b and Figure S6, Supporting Information, ∼60% of MDA‐MB‐231 or CT26 tumor cells distributed in S, G2, and M phases for control or FH nanoparticles treated cells, indicating these cells were proliferated quickly. In contrast, tumor cells in the S, G2, and M phases significantly reduced in the MTX or FHM group, and further decreased with FMD treatment (Figure 3b and Figure S6, Supporting Information). Specifically, MDA‐MB‐231 and CT26 cells in the S or G2‐M phase significantly decreased with FHM nanoparticles and FMD treatment (Figure 3b), suggesting inhibition of DNA and RNA synthesis with FHM nanoparticles and FMD treatment efficiently inhibited cell proliferation. Detections of cell apoptosis confirmed that FHM nanoparticles with FMD treatment induced the strongest cell apoptosis compared with other groups (Figure S7, Supporting Information).

### 2.3 Metabolic Analysis of Combined Therapy

To further determine the nucleotide metabolism, molecules that are crucial for DNA and RNA synthesis were extracted and detected by high performance liquid chromatography (HPLC), including 5‐methyltetrahydrofolate (5‐MTHF), guanine nucleotide (GMP), and adenine nucleotide (AMP). 5‐MTHF is the primary form of biologically active folic acid, and the donor of the methyl group that plays a vital role in nucleotide synthesis.^[^ ^43^ ^]^ As shown in Figure 3c, 5‐MTHF in control group significantly decreased when cultured with FMD medium, indicating limited nutrients decreased the production of 5‐MTHF. In tumor cells, glucose or other nutrients can be used to synthesize serine and further convert into glycine through serine hydroxymethyltransferase (SHMT), transferring a methyl group to THF to generate 5,10‐methylenetetrahydrofolate (5,10‐MTHF) and then 5‐MTHF (Figure 3d). Both 5,10‐MTHF and 5‐MTHF are necessary for nucleic acid synthesis. MTX can inhibit the function of DHFR and then affect THF to inhibit the production of 5‐MTHF (Figure 3d). Indeed, the amount of 5‐MTHF in tumor cells was further decreased after being treated with MTX (Figure 3c). The lowest concentration of 5‐MTHF was obtained in FHM nanoparticles and FMD treated cells (Figure 3c).

Histidine catabolism plays an essential function in protein synthesis with the help of enzymes like histidase and formiminotransferase cyclodeaminase (FTCD).^[^ ^44^, ^45^ ^]^ However, histidine catabolism drains the cellular pool of THF at the last step, resulting in the decreased amount of 5‐MTHF in tumor cells. To confirm this, two downstream products of histidine catabolism were used (i.e., urocanate and glutamate). As shown in Figure S8, Supporting Information, the cytotoxicity of MTX was obviously increased if co‐culturing with histidine or urocanate but not glutamate, indicating that consumption of 5‐MTHF is crucial to enhance the cytotoxicity of MTX. Of note, treatment of MTX or histidine did not affect the expression of the related enzymes in histidine catabolism. As shown in Figure S9, Supporting Information, the expressions of *HAL* gene (encoding histidine ammonia‐lyase), *FTCD* gene (encoding formimidoyltransferase cyclodeaminase), or *Amdhd1* gene (encoding amidohydrolase domain containing 1) were not significantly changed after being treated with histidine or MTX.

The decreased amount of 5‐MTHF in tumor cells wound compromises the nucleotide synthesis and cell proliferation. Indeed, GMP and AMP levels significantly decreased in tumor cells after treatment with FMD (Figure 3c). Treatment of MTX further decreased the concentrations of GMP and AMP in tumor cells. In being consistent with cytotoxicity, FHM nanoparticles and FMD treatment induced the lowest concentrations of GMP and AMP in tumor cells (Figure 3c). Collectively, these results reveal that triple inhibitions of the folate cycle significantly reduce the metabolic level of nucleotide synthesis and augment the cytotoxicity of MTX to tumor cells.

### 2.4 Enhancing Cellular Uptake and Tumor Accumulation of FHM Nanoparticles with FMD Treatment

**Figure 4:**
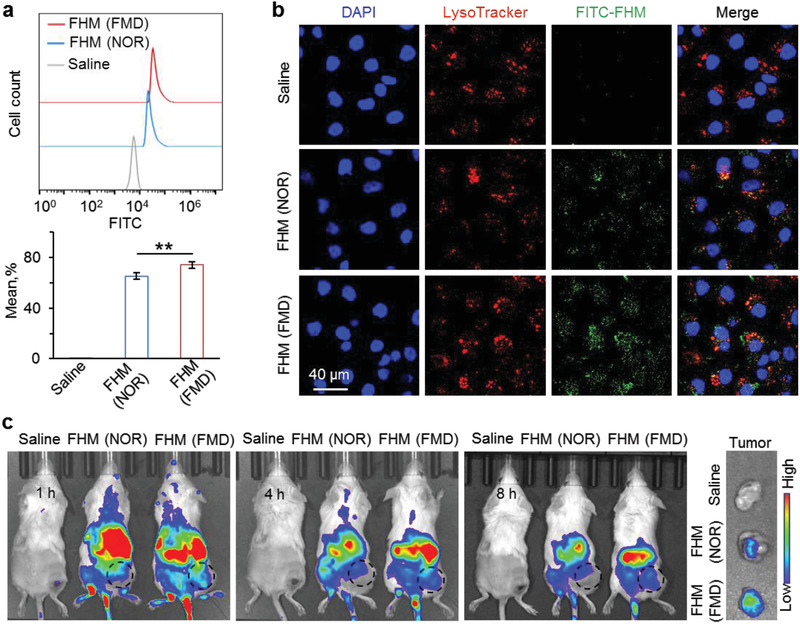
FMD treatment enhances cellular uptake and tumor accumulation of FHM nanoparticles. a) Flow cytometry and b) confocal images of FITC‐laden FHM nanoparticles treated MDA‐MB‐231 cells with or without FMD pretreatment. Error bars represent ± s.d. (*n* = 3). Significant differences were detected by one‐way ANOVA with Tukey's multiple comparisons test, **p < 0.01. c) An in vivo IVIS imaging of Cy5.5‐laden FHM nanoparticles in tumor‐bearing mice fed with typical or FMD diets. The circle dash lines indicate the tumors. The tumors were collected at 8 h for ex vivo imaging.

We investigated the cellular uptake and tumor accumulation of FHM nanoparticles by encapsulating fluorescein (FITC) or Cy5.5 dye for in vitro or in vivo imaging, respectively. Flow cytometry and confocal microscopy were used to study the cellular uptake of FHM nanoparticles. Interestingly, flow cytometry results showed that FMD treatment significantly increased the cellular uptake of FHM nanoparticles, compared to normal medium cultured cells (**Figure** **4**a). Similarly, the fluorescence intensity of FITC was stronger in FHM nanoparticles treated cells with FMD medium, indicating FMD treatment augmented the cellular uptake of FHM nanoparticles (Figure 4b).

Next, CT26 cells were subcutaneously injected at 5 × 10^5^ cells per mouse for in vivo imaging. For FMD treatment, mice were fed with a 50%‐lower‐calorie diet for one day and a 90%‐lower‐calorie diet for another day. Mice were fed a normal diet ad libitum for NOR group. As shown in Figure 4c, FHM nanoparticles efficiently accumulated at the tumor site after being intravenously injected for 1 h. Interestingly, the tumor accumulation of FHM nanoparticles were further enhanced by feeding the mice with FMD diet, compared with the NOR group. To confirm this, the tumors were collected at 8 h for ex vivo imaging. Indeed, the fluorescence intensity in FMD treated tumor is much stronger than NOR group, confirming FMD treatment enhanced the tumor accumulation of FHM nanoparticles (Figure 4c). These data show that FMD treatment can be used to enhance the cellular uptake and tumor accumulation of nanoparticles, which is crucial for nanoparticle‐mediated drug delivery.

### 2.5 In Vivo Antitumor Capacity of FHM Nanoparticles

**Figure 5:**
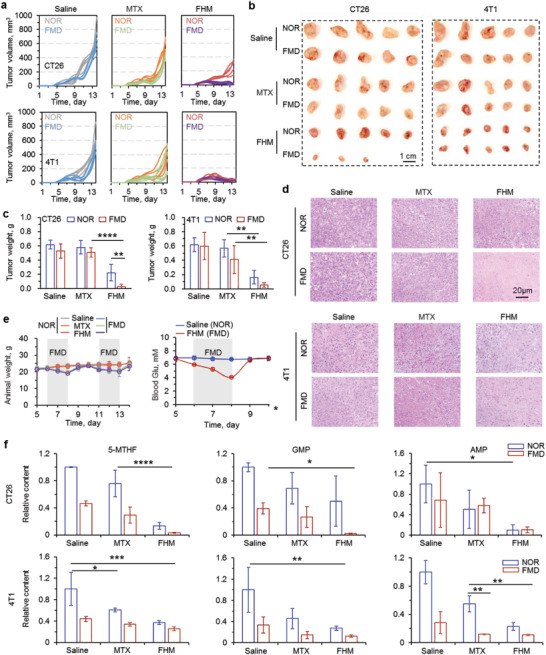
In vivo antitumor capacity of FHM nanoparticles. a) The growth curves, b) tumor images, and c) tumor weights of CT26 and 4T1 tumors in mice with various treatments. Error bars represent ± s.d. (*n* = 3). Significant differences were detected by two‐way ANOVA with Tukey's multiple comparisons test, **p < 0.01, ****p < 0.0001. d) Representative H&E images of tumors obtained from mice treated various formulations. e) The weights of mice with various treatments during in vivo studies (left) and blood glucose of saline (NOR) and FHM (FMD) treated mice from day 5 to day 10. The gray area shows the time frame of FMD treatment. f) Detections of 5‐MTHF, GMP, and AMP in both CT26 and 4T1 tumors from mice with various drug formulations. Error bars represent ± s.d. (*n* = 3). Significant differences were detected by two‐way ANOVA with Tukey's multiple comparisons test, *p < 0.05, **p < 0.01, ***p < 0.001, ****p < 0.0001.

Two murine tumor cells (i.e., CT26 and 4T1) were subcutaneously injected into BALB/c mice to assess the antitumor capacity of FHM nanoparticles without or with FMD treatment. Specifically, the mice were fed with FMD diets for three days as one cycle, and total two cycles were conducted during the experiment. For each FMD cycle, the mice were fed with 50%‐lower‐calorie diets for one day and 90%‐lower‐calorie diets for another two days. Water was available during the entire experiment period for all mice. Various drug formulations were intravenously injected into the mice on the second day of the FMD cycle. As shown in **Figure** **5**a, FMD treatment did not significantly affect the tumor growths of CT26 and 4T1 tumor models. There was a slight decrease for the mice being treated with MTX, probably due to a low dose of MTX (12.5 mg kg^−1^ body weight) employed in this study. Importantly, FHM nanoparticles with FMD treatment strongly inhibited the tumor growth compared to the other groups (Figure 5a). We further confirmed these results with tumor images and weights. Figure 5b,c illustrates that free MTX combined with FMD treatment reduced the average weight of tumors but did not significantly change. In contrast, FHM nanoparticles significantly decreased tumor weights and were further enhanced if combing with FMD treatment. Next, tumors were collected and resected for hematoxylin and eosin (H&E) staining. Similar to saline group, most of cells were variable in free MTX treated mice (Figure 5d). However, extensive necrotic cells were observed in the tumors treated with FHM nanoparticle and FMD for both CT26 and 4T1 tumors, confirming the best antitumor capacity of FHM nanoparticles with FMD treatment (Figure 5d).

During the in vivo treatments, we carefully recorded the body weights of all groups. As shown in Figure 5e, the body weight of each group decreased (∼20%) during each FMD cycle but recovered immediately after feeding with normal diets. The blood glucose in serum was also measured during the first FMD cycle. Results indicated that the blood glucose decreased once we used a 90%‐lower‐calorie diet to feed the mice, ∼40% lower than mice on the regular diet group. However, blood glucose quickly returned to normal after feeding the mice with regular diets (Figure 5e). As abovementioned, glucose or other nutrients are closely related with the production of 5‐MTHF. Next, the amounts of 5‐MTHF, AMP, and GMP in tumors with various treatments were determined by HPLC (Figure S10, Supporting Information). As shown in Figure 5f, the amount of 5‐MTHF in CT26 tumors from mice treated with FHM nanoparticles was significantly lower than the tumors treated with free MTX. Importantly, the lowest amount of 5‐MTHF was obtained in FHM nanoparticles and FMD treated tumors, indicating that the triple inhibitions of one‐carbon metabolism efficiently inhibited the production of 5‐MTHF in CT26 tumor cells. Similarly, GMP was significantly lower in FHM nanoparticles and FMD treated mice than other groups. The amount of AMP in tumors was minimal in the FHM nanoparticles group without or with FMD treatment, and significantly lower than free MTX treated tumors (Figure 5f). We observed similar trends in the 4T1 tumor model (Figure 5f). The amounts of 5‐MTHF, AMP, and GMP were lower in FHM nanoparticles and FMD treated mice than other groups (Figure 5f). These data indicated that molecules used for DNA and RNA synthesis are significantly reduced with our strategy, resulting in the enhanced antitumor capacity.

Lastly, we evaluated the potential toxicity of FHM nanoparticles and FMD treatment. As shown in Figure S11, Supporting Information, H&E staining images showed no obvious damages in major organs obtained from mice treated with FHM nanoparticles and FMD, revealing the safety of FMD as an adjuvant therapy for FHM nanoparticles. Serum biochemical indicators illustrated that the functions of liver and kidney were not significantly changed after being treated with FMD or FHM nanoparticles (Figure S12, Supporting Information). Similarly, blood biochemistry tests revealed that FMD treatment and FHM nanoparticles had minimal effects on the biofunctions of mice (Figure S13, Supporting Information). Meanwhile, the concentration of Fe ions in serum was among the normal levels after treating with FHM nanoparticles (Figure S14, Supporting Information), suggesting the release of Fe ions was minimal after being injected in mice. Indeed, in vitro analysis confirmed the sustained release of Fe ions from FHM nanoparticles, but strongly increased after being exposed to high concentrations of ROS (Figure S14, Supporting Information). Overall, our results provided evidence for the benefits and safety of FMD as a complementary method for FHM nanoparticle‐mediated cancer therapy.

## 3 Conclusions

In summary, we synthesized a unique nanoplatform by chelating histidine and MTX with ferrous ions. MTX inhibited the functions of DHFR, TYMS, and ATIC, thereby decreasing one‐carbon metabolism and de novo pyrimidine or purine synthesis. Histidine metabolism further depleted intracellular THF to block the one‐carbon metabolism. As a result, the combination of histidine and MTX significantly decreased nucleotide metabolism in tumor cells for DNA and RNA synthesis. Moreover, we utilized an FMD treatment to reduce the nucleotide metabolism, a periodic low‐calorie and low‐protein diet. Interestingly, FMD treatment significantly enhanced the cellular uptake of FHM nanoparticles in tumor cells and accumulation at the tumor site. Probably due to triple inhibitions of one‐carbon metabolism, FHM nanoparticles with FMD treatment significantly inhibited the proliferation of three kinds of tumor cells. For in vivo studies, we confirmed the antitumor capacity of FHM nanoparticles with two tumor models (i.e., CT26 and 4T1). With a 95% lower dose of MTX, FHM nanoparticles efficiently inhibited tumor growths when combined with FMD treatments. Collectively, this study laid the foundation for a therapeutic strategy that would utilize dietary modification and nanodrugs for cancer therapy.

## 4 Experimental Section

### Materials

Roswell Park Memorial Institute 1640 (RPMI 1640) medium was purchased from Gibco BRL (Germany). Sugar‐free RPMI 1640 medium was purchased from Procell (China). Penicillin‐streptomycin and Trypsin‐EDTA were purchased from Beyotime (China). L‐histidine and ascorbic acid were purchased from Sigma (St. Louis, USA). D‐glucose, MTX, and AMP chemical standards were purchased from Solarbio (China). 5‐MTHF and GMP chemical standards were purchased from Meilunbio (China) and TMRM (China), respectively. Ferrous sulfate was purchased from XiLONG Scientific (China). TBAB was purchased from J&K Scientific (China). Lower‐calorie jelly was purchased from ReadyDietech (China). HPLC grade acetonitrile and methanol were purchased from HiPureChem (China). All other chemicals were purchased from Sigma unless specifically mentioned.

### Preparation and Characterization of Nanoparticles

For FH nanoparticles or the mixture of Fe and MTX, the feeding ratios were 2:1 (histidine: ferrous sulfate) and 1:5 (MTX: ferrous sulfate), respectively. For FHM nanoparticles, MTX, histidine, and ferrous sulfate were mixed at indicated feeding ratios (MTX: histidine: ferrous sulfate). Nanoparticles were synthesized by stirring the above mixed solutions at 1500 rpm for 3 h at 95 °C. Then, the nanoparticles were collected by centrifugation and washed with deionized (DI) water for three times. For nanoparticles used for in vitro or in vivo imaging, we mixed FITC or Cy5.5 with nanoparticles at a ratio of 1:20 (FITC or Cy5.5: FHM nanoparticles) by weight. All kinds of nanoparticles were washed with DI water to remove any non‐encapsulated molecules. To confirm the size and morphology of nanoparticles, the nanoparticle solutions were dropped on a copper mesh and dried for 2 h at room temperature. The TEM images were obtained with transmission electron microscope (HT7700). For SEM imaging, nanoparticle solutions were dropped on a silicon chip and dried at room temperature for scanning images on an electron microscope. The samples were coated with a thin film of Au before imaging with Hitachi S4800. The XPS data of FHM nanoparticles were obtained with a Thermo ESCALAB 250XI X‐ray performed photoelectron spectroscopy after drying the samples by N_2_ flow. The FTIR spectra, histidine, MTX, FH and FHM nanoparticles were obtained with a spotlight 200i after drying the samples by N_2_ flow.

### Cell Viability Assays

MDA‐MB‐231, CT26, or 4T1 tumor cells were seeded in a 96‐well plate at a density of 5000 cells per well. For FMD pretreatment, the cell medium was switched from a normal RPMI 1640 medium (2 g L^−1^ of glucose and 10% of FBS) to an FMD medium (0.5 g L^−1^ of glucose and 1% of FBS) for 24 h. Afterward, different concentrations of MTX or nanoparticles were added into the normal or FMD medium. We incubated the tumor cells with various drug formulations for 48 h, then detected cell viability by adding 3‐(4, 5‐dimethylthiazol‐2‐yl)‐2,5‐diphenyltetrazolium bromide (MTT) to synthesize formazan. Plate Reader was used to detect the optical density of formazan solution re‐dissolved by DMSO at a wavelength of 490 nm. The authors calculated cell survivals by comparing treated cells to non‐treated ones. Cell viability assays were conducted in triplicate for each sample in all experiments.

### Cell Cycle and Apoptosis Analysis by Flow Cytometry

Tumor cells were seeded in a 6‐well plate at an appropriate cell density. For FMD group, we performed the pretreatment as described above. Free MTX or nanoparticles were incubated with tumor cells for 24 h. Then, the cells were collected, washed with phosphate buffered saline (PBS), and resuspended in 70% absolute ethanol for 4 h. The samples were further washed with PBS before incubating with propidium iodide and RNase for 30 min at 37 °C. Cell cycle analyses were performed using a BD Accuri C6 flow cytometer. For cell apoptosis analysis, tumor cells were incubated with free MTX or FHM nanoparticles for 48 h. Then, tumor cells were collected, washed with PBS, and stained with Annexin V‐FITC and propidium iodide for 15 and 5 min, respectively. Cell cycles were performed using a BD Accuri C6 flow cytometer.

### Quantitative Real‐Time PCR

Tumor cells were washed with PBS and incubated with Trizol to obtain RNA. The reverse transcriptase reaction was performed using Hifair III (Yeasen Biotechnology). Quantitative PCR reactions were performed with SYBR green reagent (Yeasen Biotechnology) according to the manufacturer's instructions. The QuantStudio 6 Flex (Applied Biosystems) qPCR instrument was used for reactions. Primers used for *FTCD* forward primer: AACCTGCTAAGCACCAAGGA; reverse primer: GGACCTTTTTCAGACGTCCA. *HAL* forward primer: GGTGGCCTTAGAGGACAATG; reverse primer: GCTCCCG GTATTTGCTGTAG. *Amdhd1* forward primer: TCCACGAGTTTGCAATGAAG; reverse primer: CTCCACCGTGAAGTTGATCC.

### Cellular Uptake of Nanoparticles

For flow cytometry analysis, MDA‐MB‐231 cells were seeded in a 6‐well plate with FMD pretreatment before culturing in an FMD medium containing 5 µg mL^−1^ FITC encapsulated in nanoparticles for 4 h. Then, the cells were collected, washed with PBS three times, and analyzed as with the cell cycle analysis. For in vitro cell imaging, MDA‐MB‐231 cells were seeded in a confocal dish and treated as above. Tumor cells were washed three times with PBS before being stained with lysotracker green for 2 h. Finally, tumor cells were fixed with 10% paraformaldehyde for 20 min at room temperature, and the nucleus were stained by 4′,6‐diamidino‐2‐phenylindole (DAPI). The confocal imaging was conducted with Laser scanning confocal microscopy (Z‐760).

### In Vivo Distribution of FHM Nanoparticles

All animal experiments in this study were performed in accordance with the relevant rules and regulations approved by Institutional Animal Care and Use Committee of National Center for Nanoscience and Technology (NCNST21‐2103‐0403). We subcutaneously injected CT26 cells (5 × 10^5^) into the right dorsal side of the hind limb of BALB/C mice and injected saline (100 µL) or Cy5.5‐laden FHM nanoparticles (50 µg dissolved in 100 µL saline) via caudal vein on the second day of FMD treatment. All the mice groups were imaged at 1, 4, and 8 h post‐injection with IVIS imaging system. The mice were euthanized after being imaged at 8 h, and tumors were collected for ex vivo imaging. For detecting ferrous ions released from FHM nanoparticles in vivo, FHM nanoparticles or saline were intravenously injected into the mice. Serums were collected from mice at 1, 8, and 24 h and centrifuged at 10 000 rpm for 15 min to remove circulated nanoparticles. The samples were diluted and tested by an inductively coupled plasma mass spectrometer (NexION 300X).

### In Vivo Antitumor Capacity and Safety

We subcutaneously injected 4T1 cells (5 × 10^5^ per mouse) into the left dorsal side of the hind limb and CT26 cells (5 × 10^5^ per mouse) into the right dorsal side of the hind limb on each eight‐week mouse. A total of 30 mice with similar tumor sizes comprised six groups: saline (NOR and FMD treatments), free MTX (NOR and FMD treatments), and FHM nanoparticles (NOR and FMD treatments). For each FMD treatment, mice were fed with a 50%‐lower‐calorie diet for one day and a 90%‐lower‐calorie diet for the next two days. The free MTX and FHM nanoparticles were injected via caudal vein on the second day of each FMD treatment at a dose of 12.5 mg kg^−1^ body weight. The blood glucose was recorded during FMD treatment. Animal weight and tumor volume were measured daily. The tumor volume (*V*) was calculated as *V* = *L* × *W* ^2^ × 0.5, where *L* denoted the long diameter, and *W* denoted the short diameter determined using a caliper.^[^ ^46^ ^]^ All mice were euthanized at day 14. Hearts, livers, spleens, lungs, and kidneys were collected and fixed in a 4% paraformaldehyde for H&E staining. Tumors were obtained and randomly picked for H&E staining or the detection of specific metabolites.

### Metabolite Extraction and Detection

The protocols for metabolites extraction were used as previously described, but with slight changes.^[^ ^47^ ^]^ To extract intracellular metabolites GMP and AMP, 2 × 10^6^ cells were collected, washed by PBS, and resuspended in an extraction buffer of 80% methanol and 20% H_2_O. Then, the samples were vortex‐oscillated for 10 s and centrifuged at 18 000 g at 4 °C for 15 min. The supernatant was transferred to a new tube, dried by N_2_ flow, and resuspended in DI water before detection by HPLC.

For 5‐MTHF detection, the same quantity of cells was collected, washed by PBS, and resuspended in an extraction buffer of 80% methanol and 20% H_2_O containing 25 mm sodium ascorbate. Afterward, the authors dried the metabolites by N_2_ flow suspended in a reconstitution buffer, containing 0.5% ascorbic acid, 1% K_2_HPO_4_, 0.5% 2‐mercaptoethanol, and 20% rat serum. Sodium ascorbate and 2‐mercaptoethanol were the antioxidants to protect 5‐MTHF from oxidation. In addition, the rat serum provided enzymes to strip the tail of polyglutamate. Metabolic extraction resuspended in reconstitution buffer were incubated at 37 °C for 2 h and centrifuged at 18 000 g at 4 °C for 15 min. Eventually, the supernatant was collected for further detection.

Tumors obtained from mice were frozen in liquid nitrogen immediately and homogenized by a tissue homogenizer at 4 °C in an extraction buffer. Then, the tumor homogenates were centrifuged at 18 000 g for 15 min at 4 °C. Finally, the supernatant was collected, dried, and resuspended as described.

Metabolite detection was studied using a high‐performance liquid chromatography Prominence LC‐20A (Shimadzu) with a Hypersil GOLFD HPLC C18 column (150 × 4.6 mm, 5 µm particle size, Thermo Fisher). The following buffers were used to detect small metabolites GMP and AMP: buffer A, 5 mm TBAB in pH 7.4 phosphate buffer; buffer B, methanol. The detection wavelength was 254 nm and chromatographic gradient was conducted by running 10% buffer B at a speed of 0.5 mL min^−1^. For folate species 5‐MTHF detection, 0.05 mm KH_2_PO_4_ was used as buffer A and acetonitrile was used as buffer B. The chromatographic gradient was used 6% of buffer B running at 1 mL min^−1^ and the detection was performed at 290 nm.

### Statistical Analysis

All data were presented as mean ± s.d. from at least three independent experiments. Comparisons among more than two groups were performed by one‐way or two‐way ANOVA. Statistical analyses were performed with GraphPad Prism software 8.0, and a statistical significance was accepted at *p* value of less than 0.05. *p < 0.05, **p < 0.01, and ***p < 0.001.

## Conflict of Interest

The authors declare no conflict of interest.

## Supporting information

Supporting Information

## Data Availability

The data that support the findings of this study are available from the corresponding author upon reasonable request.
