# Supplementary material for: Regulation of Nucleotide Metabolism with Nutrient‐Sensing Nanodrugs for Cancer Therapy
Source: Adv Sci (Weinh). 2022 May 4;9(20):2200482. doi: 10.1002/advs.202200482 (PMC9284143; doi:10.1002/advs.202200482)
Supplement: Supplementary file 1 — Supporting Information [file ADVS-9-2200482-s001.pdf]

Supporting Information

Regulation of Nucleotide Metabolism with Nutrient-sensing Nanodrugs for Cancer Therapy

Xinye Wang[1,3†], Wen Su[1†], Yongbin Jiang[2†], Fuhao Jia[1], Wenping Huang[1], Jie Zhang[1], Yue Yin[1] and Hai Wang[1,3]*

[1]CAS Key Laboratory for Biomedical Effects of Nanomaterials & Nanosafety, CAS Center for Excellence in Nanoscience, National Center for Nanoscience and Technology, 100190 Beijing, China
[2]Zhangjiakou First Hospital, 075000 Xhangjiakou, China
[3] University of Chinese Academy of Sciences, 100049 Beijing, China

*Correspondence should be addressed to:
E-mail: wanghai@nanoctr.cn

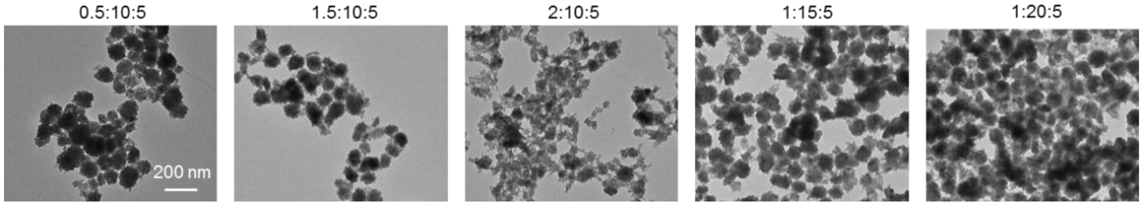

Figure S1. TEM images of FHM nanoparticles with various feeding ratios of MTX, histidine and ferrous sulfate. TEM image of FHM nanoparticles with a feeding ratio of 1:10:5 was given in **Figure 2a**.

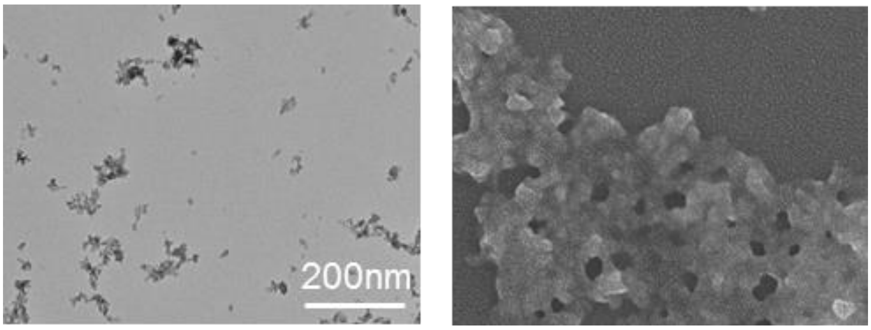

Figure S2. TEM and SEM images of materials formed with MTX and ferrous ions. No obvious nanoparticles are observable in TEM (left) or SEM images (right).

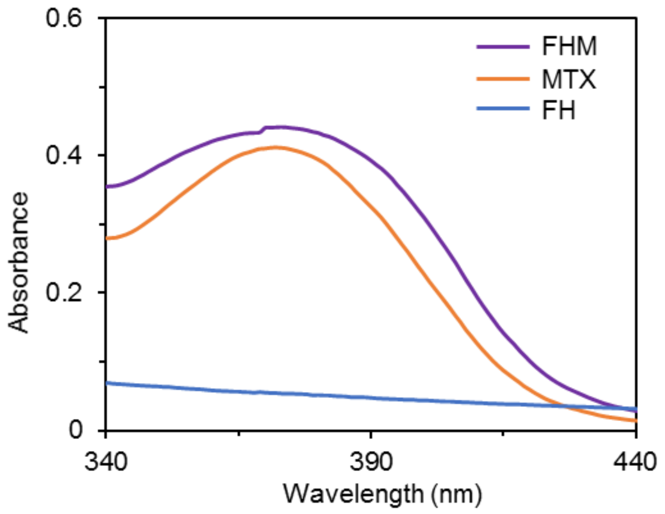

Figure S3. UV-Vis absorbance of FH, MTX and FHM nanoparticles. The absorbance peak of MTX can be detected in the FHM nanoparticles, suggesting the successful encapsulation of MTX.

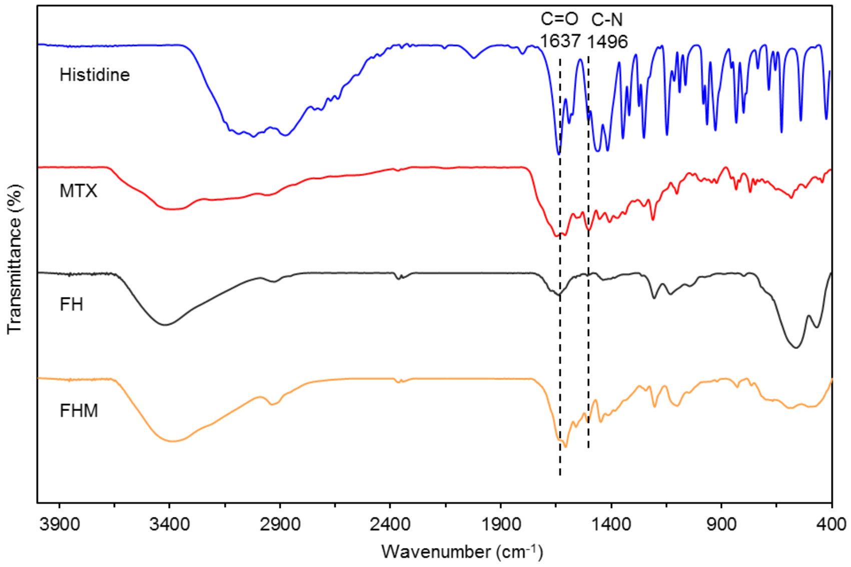

Figure S4. FTIR spectra of histdine, MTX, FH or FHM nanoparticles. The absorbance bands identified at 1637 cm^{-1} of histidine and MTX referred to the carboxyl groups shift to 1608 cm^{-1}, which indicated a strong intramolecular coordination bonding between carboxyl group and ferrous irons. Imidazole absorbance band of histidine in 1496 cm^{-1} shifted to 1504 cm^{-1} in histidine, indicating the formation of strong intramolecular coordination between imidazole and ferrous irons.

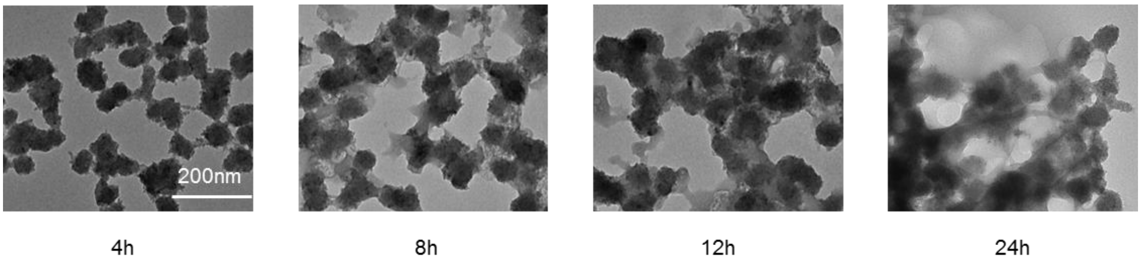

Figure S5. TEM images of FHM nanoparticles after exposed to ROS showing the ROS-responsiveness of FHM nanoparticles.

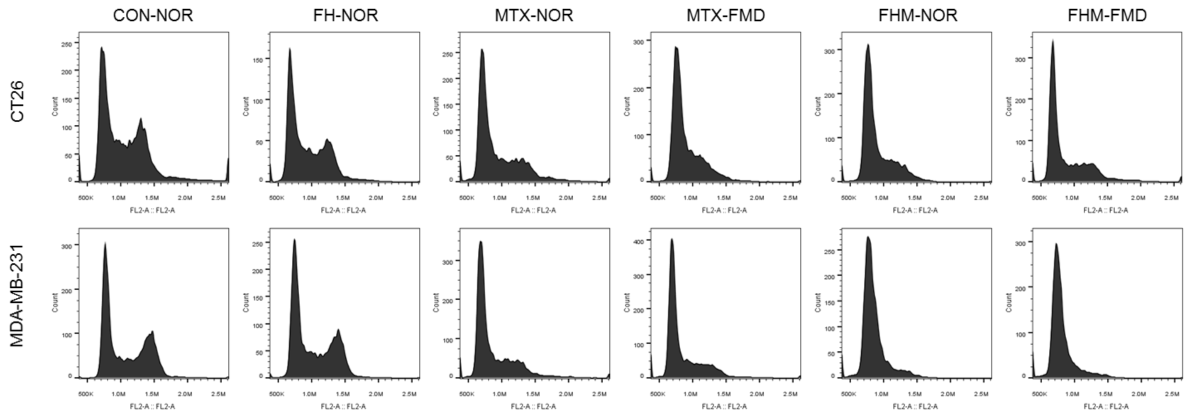

Figure S6. The representative flow cytometry images of tumor cells with various treatment as shown in **Figure 3b**.

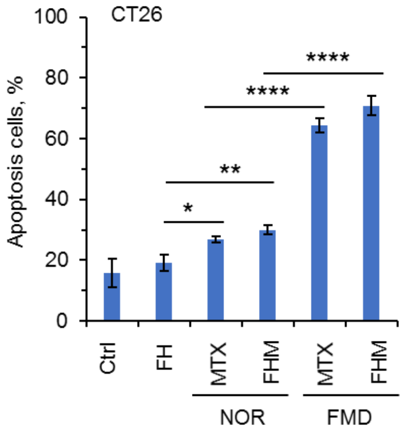

Figure S7. Cell apoptosis of CT26 tumor cells treated with FH nanoparticles, free MTX or FHM nanoparticles for 48 h with or without FMD treatment at an MTX concentration of 16 μg ml^{-1}. Error bars represent ± s.d. (n = 3). Significant differences were detected by one-way ANOVA with Tukey's multiple comparisons test, *$p < 0.05$, **$p < 0.01$, ***$p < 0.001$, ****$p < 0.0001$.

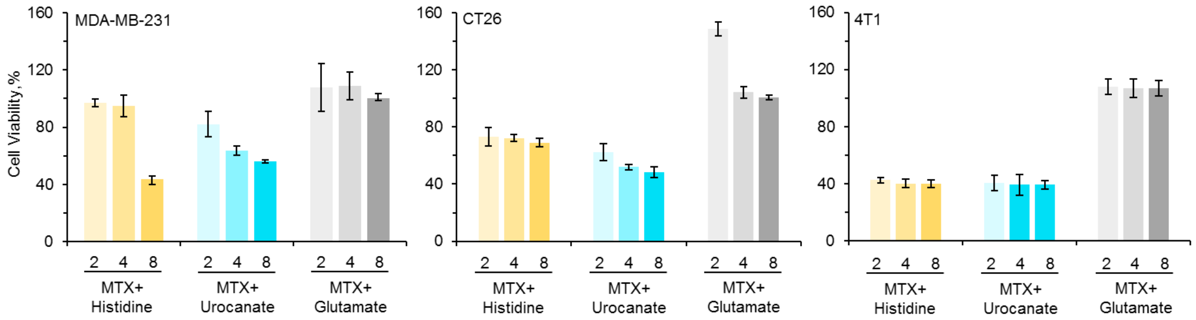

Figure S8. Cell viability of MDA-MB-231, CT26, and 4T1 tumor cells treated with the combination of MTX and 10 µM histidine, urocanate or glutamate. Error bars represent ± s.d. (n = 3).

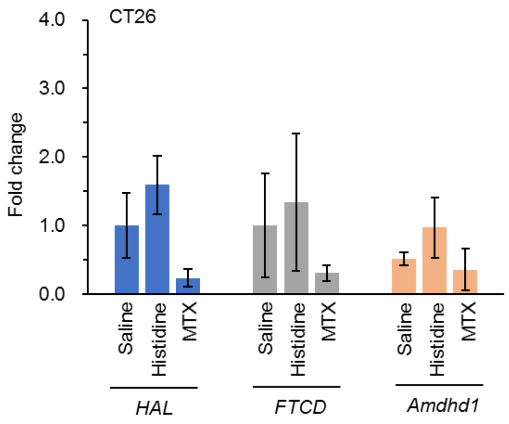

Figure S9. Expression of *HAL*, *Amdhd1* and *FTCD* genes in CT26 cells treated with 10 µM histidine or MTX. Error bars represent ± s.d. (n = 3).

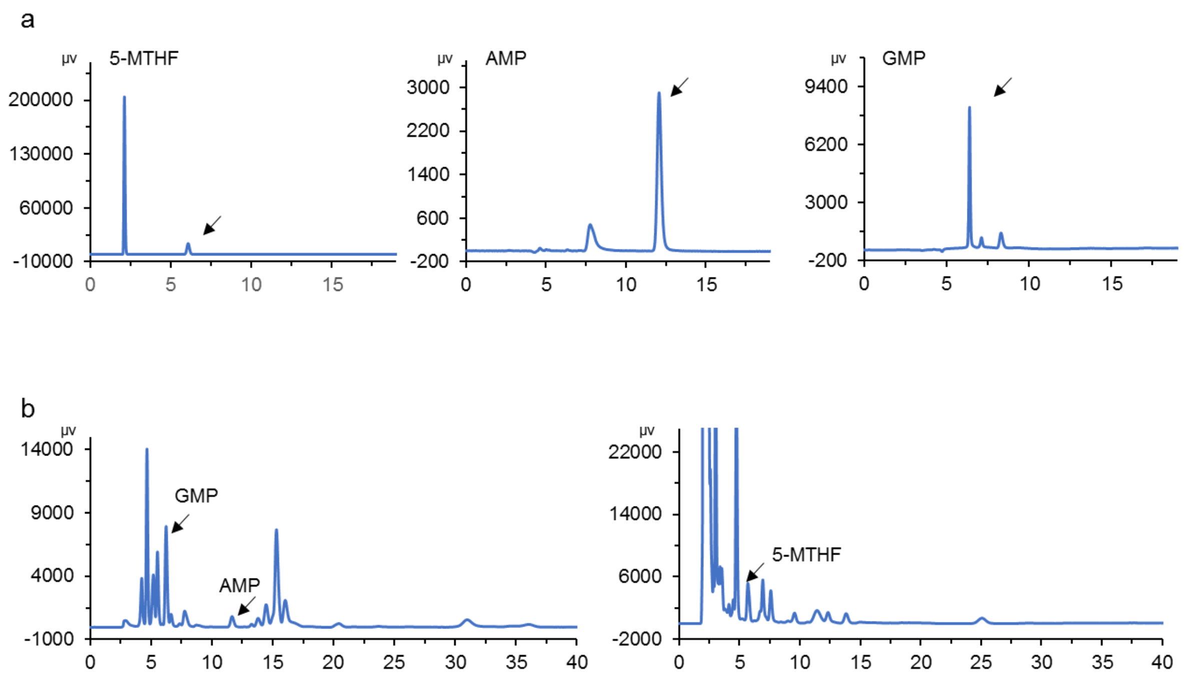

Figure S10. (**a**) The representative peak of 5-MTHF, AMP and GMP determined by HPLC (indicated by the arrows). (**b**) The data of the sample collected from tumor and the indicated peaks were used for calculation.

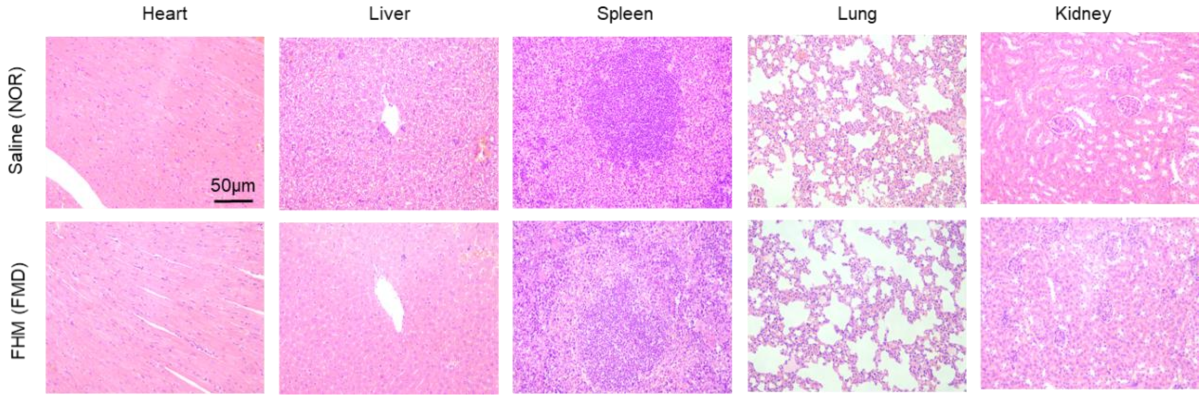

Figure S11. H&E images of major organs (heart, live, spleen, lung and kidney) collected from *in vivo* study. Compared with saline treated mice, FHM nanoparticles combined with FMD treatments did not cause obvious damages to the major organs.

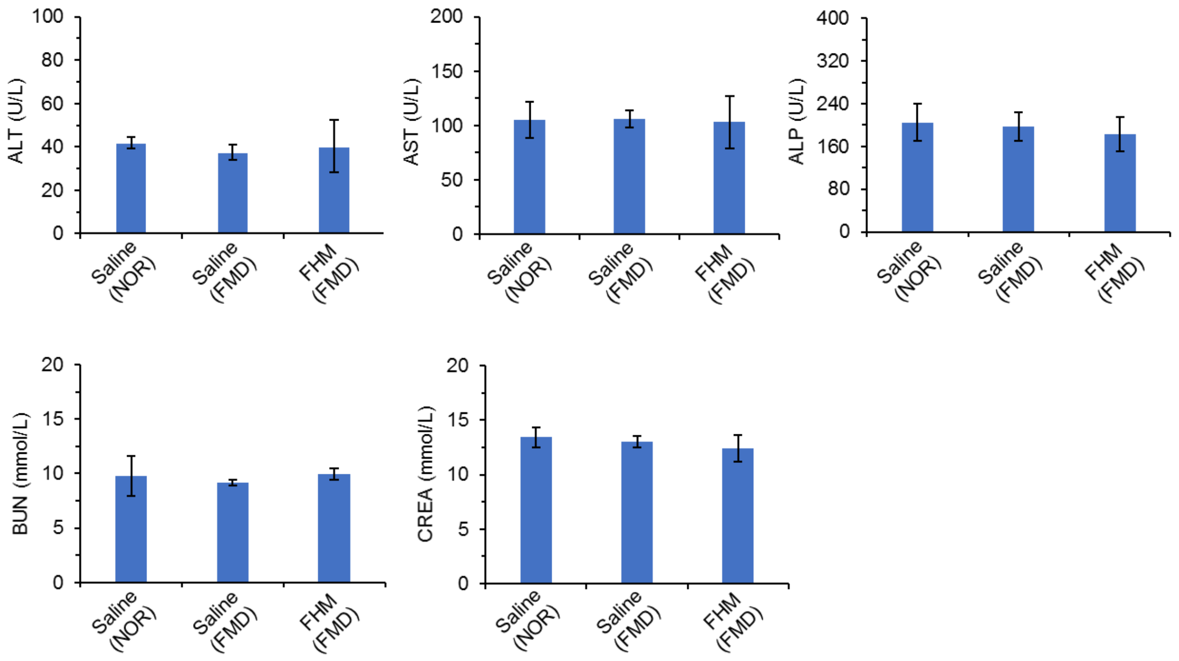

Figure S12. Evaluation of liver function of mice treated with FMD or FMD with FHM nanoparticles by measuring alanine aminotransferase (ALT), aspartate aminotransferase (AST) and alkaline phosphatase (ALP). Kidney function of mice with various treatments was determined by measuring urea nitrogen (BUN) and creatinine (CREA). No statistical difference was observed among various groups. Error bars represent ± s.d. (n = 3).

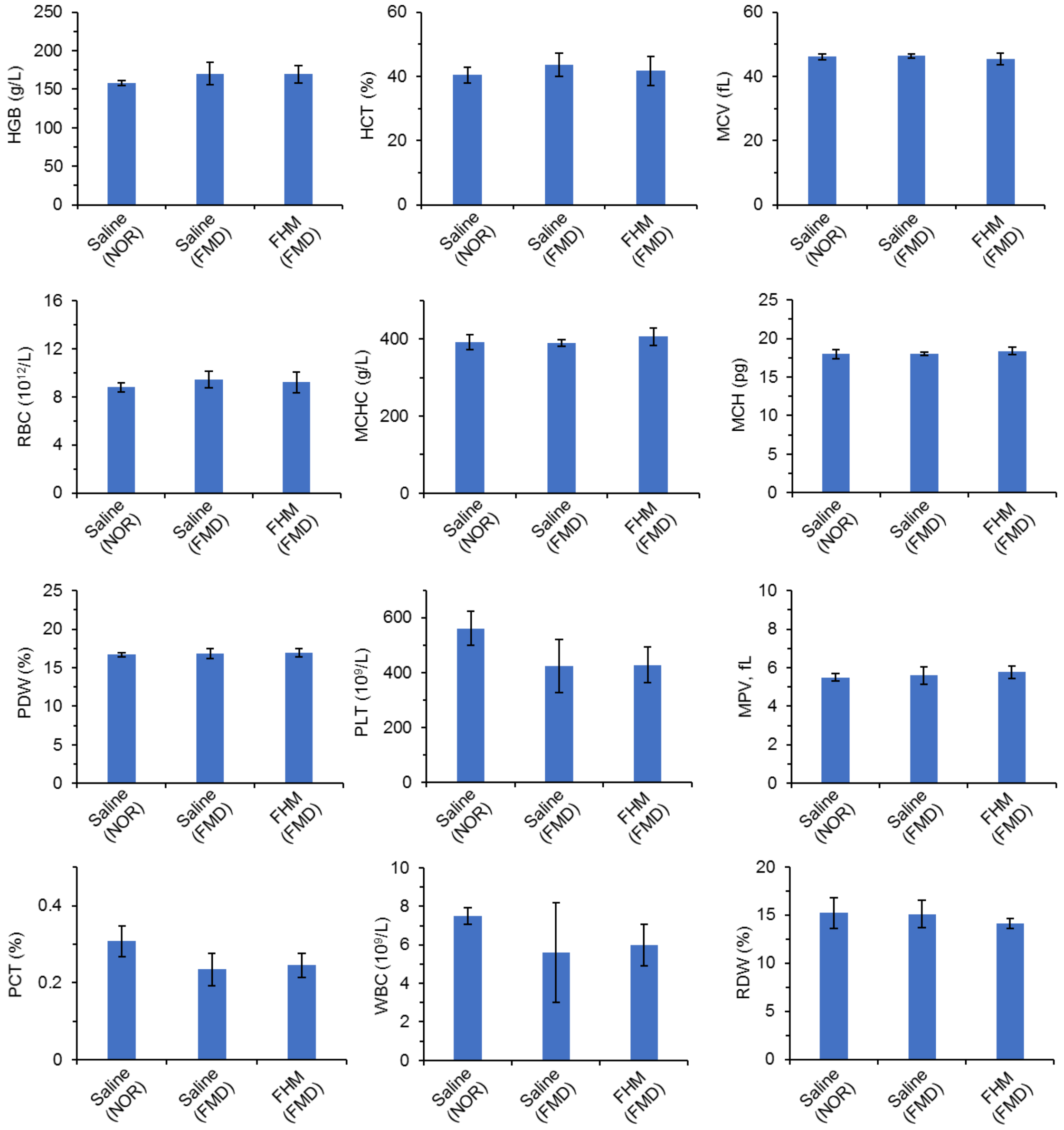

Figure S13. Detections of hemoglobin (HGB), hematocrit (HCT), mean corpuscular volume (MCV), red blood cells (RBC), mean corpuscular hemoglobin concentration (MCHC), mean corpuscular hemoglobin (MCH), red cell distribution width (RWD), platelet count (PLT), mean platelet volume (MPV), platelet distribution width (PDW), white blood cell (WBC) and plateletcrit (PCT) in blood collected from mice with various treatments. Error bars represent ± s.d. (n = 3).

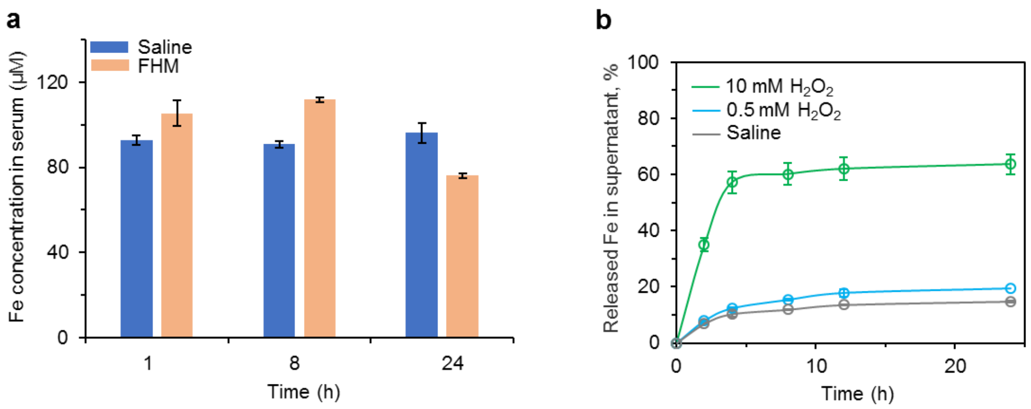

Figure S14. (a) The concentration of ferrous ions in mouse serum after injected with saline or FHM nanoparticles at a dose of 12.5 mg kg^{-1} body weight. Error bars represent ± s.d. (n = 3). (b) The sustained release of ferrous ions from MTX in saline or low concentration H_2O_2 solution. In contrast, ferrous ions quickly released from FHM nanoparticles in high concentration of H_2O_2 solution. Error bars represent ± s.d. (n = 3).
